# Fostering interprofessional identity formation to support interprofessional collaboration – Identifying guidelines for educational design

**DOI:** 10.1007/s10459-025-10478-9

**Published:** 2025-10-20

**Authors:** Annemarie B. Sänger, Renée E. Stalmeijer, Simon Beausaert, Jascha de Nooijer

**Affiliations:** 1 School of Health Professions Education, Faculty of Health, Medicine and Life Sciences, Maastricht University, Maastricht, The Netherlands; 2 Educational Research and Development, School of Business and Economics, Maastricht University, Maastricht, The Netherlands

**Keywords:** Interprofessional identity, Interprofessional identity formation, Interprofessional collaboration, Curriculum design, Interprofessional education, Higher education

## Abstract

Undergraduate interprofessional education aims to prepare health professions students for a future work field characterised by wicked problems demanding interprofessional collaboration (IPC). Interprofessional identity (IPI) has been suggested as key to IPC, but how to foster its formation remains underdeveloped. Therefore, we aimed to identify (1) the main characteristics of IPI and (2) guidelines for educational design fostering interprofessional identity formation (IPIF). Per critical review methodology, we analysed educational sciences, health professions education and management sciences literature. The results of two iterative, non-exhaustive literature searches were analysed through reflexive thematic analysis. The first search focused on conceptualisations of IPI to identify its main characteristics and theories explaining IPIF, the second on the mechanisms of these theories fostering IPIF to derive guidelines for educational design. Analysis of the first search yielded five themes characterising IPI: (1) sense of belonging to an interprofessional team; (2) commitment to working interprofessionally; (3) values, attitudes, beliefs, and ethics related to IPC; (4) knowledge and understanding of roles, responsibilities, and expertise; and (5) IPC skills. We identified five prevalent theories explaining IPIF. Analysis of the second search resulted in guidelines for educational design fostering IPIF targeting the student, interprofessional team, faculty, and curriculum. Fostering IPI alongside IPC competencies is crucial for preparing students for IPC. This critical review highlighted key characteristics of IPI and proposes ED-IPIF, a theory-based framework for fostering IPIF through integrated educational design. Future research should address existing gaps and empirically test the ED-IPIF through longitudinal studies and robust tools for measuring IPI.

**Supplementary information:**

The online version contains supplementary material available at 10.1007/s10459-025-10478-9.

## Introduction

Interprofessional education (IPE) at the undergraduate level aims to prepare health professions students for future collaborative work in interprofessional teams within a work field that, as Horn (2023) notes, is increasingly characterised by complex and multidisciplinary problems. These problems, often referred to as “wicked problems”, are unique and lack a clear origin. They involve shifting and often contradictory requirements, and have no single solution (Rittel & Webber, 1973). According to Head (2008, 2022) and Alford and Head (2017), wicked problems vary from low to high in their levels of complexity (e.g., interdependent elements); uncertainty (e.g., unpredictable consequences); and divergence (e.g., conflicting stakeholder perspectives). Given this variability, no one-size-fits-all approach can effectively tackle them (Alford & Head, 2017).

Wicked problems within the context of healthcare do not only relate to patient care, but also to systemic and societal issues (Keijser et al., 2020). Healthcare challenges that can be discussed as wicked problems include workforce shortages, global disease management (e.g., HIV/AIDS), and chronic disease prevention and management (World Health Organization, 2010). Tackling such wicked problems requires the integration of expertise from multiple disciplines. This demands collaboration beyond the boundaries of individual professions (Horn, 2023; Rittel & Webber, 1973), and extending beyond healthcare. In healthcare settings, this collaborative approach is known as interprofessional collaboration (IPC) (World Health Organization, 2010), and is suggested as a strategy to address the healthcare sector’s most complex challenges.

Through IPC, professionals integrate diverse expertise from the professions and disciplines involved to generate new insights that no single profession can provide (Reinders & Pesut, 2022). In this paper, we describe IPC as collaboration between members of different professions that is characterised by shared goals, responsibilities, accountability, and interdependence (D’Amour et al., 2005; Miettinen et al., 2024). Within healthcare, IPC is commonly defined as occurring when “multiple health workers from different professional backgrounds provide comprehensive services by working with patients, their families, carers and communities to deliver the highest quality of care across settings” (World Health Organization, 2010, p. 13). Thus far, research in health professions education has mainly focused on preparing students for IPC around patient care, not yet including the complex and multidisciplinary problems involving systemic and societal dimensions.

Despite its predicted benefits to effective patient care and tackling wicked problems, evidence from practice shows that IPC is not self-evident and faces multiple challenges. For example, IPC is impeded by uniprofessional education, resulting in a lack of understanding between professions (Jones et al., 2022; Khalili & Orchard, 2020; Wood et al., 2022). Misconceptions, stereotypes, prejudices, and superior attitudes also pose barriers to IPC, reinforcing systemic power hierarchies (Dolan & Nowell, 2023; Khalili et al., 2013; Polansky et al., 2023). Power imbalances, biases and stereotypes can also result from in-profession favouritism and out-profession discrimination (Allport, 1954; Tajfel & Turner, 1986), commonly associated with strong professional identities (Khalili & Orchard, 2020). Additionally, IPC is challenging as it requires collaboration across multiple domains. This can introduce competing interests that must be addressed (Horn, 2023; Rittel & Webber, 1973).

IPE has been suggested as a strategy to foster IPC (Khalili et al., 2019), to optimally prepare health professions students for the wicked problems that characterise their future field of work. IPE aims to equip health professions students with competencies for effective collaboration across professional boundaries within various contexts. Furthermore, IPE aims to foster students’ commitment to working interprofessionally (Tong et al., 2021). However, solely focusing on competence development during IPE may not be enough.

In the last two decades, scholars have increasingly advocated for aligning professional identity formation with competence development in curricula (Jarvis-Selinger et al., 2012; ten Cate et al., 2024; Varpio et al., 2025). This alignment is critical, as professional practice may falter when competence is misaligned with identity elements, such as beliefs and values (Jarvis-Selinger et al., 2012). Similarly, Wood et al. (2022) argue that interprofessional knowledge and skills alone are unlikely to ensure successful IPC without some alignment with interprofessional values, beliefs, attributes and behaviours. Therefore, fostering interprofessional identity (IPI) has been suggested as a way to ground and guide the IPC competencies already being taught, shifting the focus from exclusively “doing” to also “being” an interprofessional professional (Flood et al., 2019; Jarvis-Selinger et al., 2012). In line with this, Ganotice (2023) argues that IPI fosters interprofessional behaviours; accordingly, it likely enhances IPC.

Despite the recent increase in interest in interprofessional identity formation (IPIF), how IPI is defined and conceptualised varies. This is partially due to the different theoretical roots based on which the phenomenon has been explored, ranging from theories originating from psychology to sociocultural learning theories. In addition, although researchers seem to agree on the importance of IPIF, there is no clear framework addressing how to foster IPIF in education, where team-based IPC plays a central role. Therefore, this study aims to identify (1) the main characteristics of an IPI, and (2) guidelines for educational design fostering IPIF.

## Methods

### Approach

We adopted critical review methodology to address our research aims. A critical review is a type of narrative review that integrates literature and theory from various fields to develop a new approach, understanding, or framework for thinking about a certain phenomenon (Kahlke et al., 2023b). We drew on the fields of educational sciences, health professions education and management sciences to enhance our understanding of IPI's formation, its conceptualisations and explanations, and to derive corresponding guidelines for educational design. These fields were selected because the literature on IPI and IPIF is predominantly published there. We conducted two separate literature searches and analyses to address our two research aims.

### Literature search and appraisal

To identify the main characteristics of IPI, we did a literature search to capture current conceptualisations of IPI to determine commonalities across definitions, elements, dimensions, and theoretical explanations of its formation. The search comprised multiple cycles, in keeping with the typically cyclical and iterative process of critical reviews (Kahlke et al., 2023a). Included manuscripts were published between the inception of their respective databases and 1 April 2024, the date on which the search was last updated. Manuscripts originated from PubMed, ERIC, PsycARTICLES, PsycINFO, SAGE, Wiley Online Library, JSTOR, and Taylor & Francis, along with manuscripts identified through bibliography review. Search terms included “interprofessional identity”, “interprofessional identity formation”, and “interprofessional identity development”.

To identify guidelines for educational design fostering IPIF, a similar separate search strategy was undertaken to create an overview of the literature on the mechanisms of the theories commonly used to explain IPIF, as identified within the first search. The two searches differed only in their use of search terms, which in this search were the names of the theories resulting from the first search. The second search supplemented the information on IPIF from the first search.

Per critical review methodology (Kahlke et al., 2023a, 2023b), both literature searches focused on identifying relevant articles rather than being exhaustive. The search strategies were not intended to be systematic or reproducible. The first author (A.B.S.) took the lead in the search process, while other team members reviewed the identified articles and suggested additional literature. Citations were stored in EndNote, and duplicates were removed. Next, the titles and abstracts of the remaining articles were reviewed for relevance to the eligibility criteria. Included articles were full-text English articles that, for the first aim, focused on IPI, IPIF and professional identity and its formation, and, for the second aim, addressed concepts and principles of theories explaining IPIF. Full-text screening of remaining articles continued until sample sufficiency was achieved. Works that offered the most influential evidence or arguments, that is, those that were referenced often in the fields concerned, were prioritised, while we ensured that the selected sample of articles was representative of the broader literature field, as is common in critical reviews (Kahlke et al., 2023a, 2023b). Sample sufficiency was achieved when new searches yielded familiar concepts, indicating a comprehensive understanding of the subject matter (Kahlke et al., 2023a).

### Analysis

Separate analyses were performed for the first and second literature searches, led by the first author (A.B.S.). The other team members provided their reflections throughout the analytical process. Following Kahlke et al.’s (2023a) suggestion to use qualitative research analysis methods, we employed Braun and Clarke’s six-phase approach to reflexive thematic analysis (Braun & Clarke, 2021). Accordingly, to identify the main characteristics of IPI, we first familiarised ourselves with the literature on the conceptualisations of IPI that resulted from our search. This included creating an overview of the theories most commonly used to explain IPIF, which was only used to inform our second search. Second, we methodically reviewed the literature to define initial codes reflecting the commonalities among the conceptualisations. Next, initial themes were generated from these clusters, focusing on identifying possible characteristics of IPI. In the fourth phase, we developed and reviewed themes based on the initial codes, resulting in themes that each represented a characteristic of IPI. Subsequently, our themes were refined, defined and named to fully capture each identified characteristic. The outcomes of the analysis are reported in the results section.

To identify guidelines for educational design fostering IPIF, a similar approach was undertaken. However, the focus of the search was on creating an overview of the literature on the theories most commonly used to explain IPIF and their mechanisms relevant to IPIF. Thematic analysis was used to understand how these mechanisms enable the formation of the previously identified characteristics of IPI. This resulted in the definition of our themes, that is, the guidelines for educational design fostering IPIF. In the results section, these guidelines are presented as a framework for educational design.

### Reflexivity

In this reflexivity section, we reflect on how our individual experiences, backgrounds and areas of expertise shaped the approach and findings of this critical review. We adopted a constructivist stance, leveraging our team’s insights and expertise during the review process rather than seeking to produce a generalisable truth or perfectly replicable methods (Kahlke et al., 2023b). The research team consisted of four authors with a broad range of roles and experience in health professions education (A.B.S., R.E.S. and J.d.N.) and business education (S.B.). The lead author, A.B.S., is a health and human movement scientist with teaching roles in health professions education; this review is the first study in A.B.S.’s PhD research into how to foster IPIF in IPE to support IPC. Next, R.E.S. is an educational scientist by training who focuses her research, education and consultancy on workplace learning and supervision in the undergraduate and post-graduate medical education curriculum. The third team member, S.B., is a professor of learning and development in organisations, whose research focuses on how to support lifelong learning and how to set up strategic human resource development policies to support work-related learning. Lastly, J.d.N. is a professor of interprofessional teaching and learning who initiates, develops, implements and evaluates activities and research for students and teachers that include an interprofessional component.

All authors have experience with multiple types of review methodology. R.E.S. specifically is experienced in conducting critical reviews. Our author team is linked by a mutual interest in IPE design and workplace learning, and specifically fostering IPIF in IPE. Our diverse backgrounds represent the different fields we aim to bring together with this critical review, which facilitated the integration of literature from the educational sciences, health professions education and management sciences, including identifying relevant works from the field. As a team, we regularly engaged in team discussions, using our perspectives to analyse the findings and challenge assumptions to guide the review process.

In the literature, the process of acquiring an IPI is referred to as interprofessional identity formation or development. Given our constructivist stance and in keeping with the sociocultural literature exploring professional identity (Monrouxe & Rees, 2015), we use the term “formation” instead of “development”.

## Results

In the following section, we present the main characteristics of an IPI through five themes, followed by an overview of the five theories commonly used to explain IPIF. Next, we present the mechanisms that each theory suggests may foster IPIF, yielding our proposed guidelines for educational design.

### Conceptualising interprofessional identity

The following author teams explicitly conceptualised IPI: Cantaert et al. (2022); Hammick et al. (2009) as cited in Thistlethwaite et al. (2016) and Floyd and Morrison (2014); Khalili et al. (2013, 2019); Reinders (2018), Reinders and Krijnen (2023), Reinders et al. (2018); Thistlethwaite et al. (2016), which was based on the report from the Interprofessional Education Collaborative Expert Panel (2011); Tong et al. (2020); and Wood et al. (2022). Online Resource 1 provides an overview of the conceptualisations used.

We identified five common themes across all conceptualisations, sorted by prevalence: (1) sense of belonging to an interprofessional team; (2) commitment to working interprofessionally; (3) values, attitudes, beliefs, and ethics related to IPC; (4) knowledge and understanding of roles, responsibilities and expertise; and (5) IPC skills. Table 1 provides an overview of the themes, their definitions, related concepts, and definitions by the author teams, as identified in our corpus.

**Table 1:** Definition of interprofessional identity themes

Theme	Described in this article as	**Definition provided by authors**^**a**^	Our resulting theme definition
**Sense of belonging to an interprofessional team**	Khalili et al. (2013) *and* Khalili et al. (2019) Dual identity	Dual identity = “the development of robust sense of belonging to both own profession (in-profession favouritism) and to the interprofessional community (interprofessional favouritism) in which individuals view themselves simultaneously as a member of their own profession and the interprofessional team/community” (Khalili et al., 2019, p. 28). Khalili et al. (2013) *provided no definition.*	A sense of belonging to an interprofessional team, where individuals view themselves as a member of an interprofessional team consisting of individuals from different professions that pursue joint goals
	Tong et al. (2020) Interprofessional identity	Interprofessional identity = “a robust cognitive, psychological and emotional sense of belonging to an interprofessional community(s), needed to achieve context-dependent goals” (Tong et al., 2020, p. 6).	
	Reinders (2018), *Reinders and Krijnen (*2023*), and* Reinders et al. (2018) 1. Interprofessional identity 2. Interprofessional belonging	1. Interprofessional identity = “the degree to which an individual feels a part of and is committed to a group or social category consisting of different professions that pursue joint goals” (Reinders & Krijnen, 2023, p. 2). 2. Interprofessional belonging = “related to social inclusiveness of one’s own profession as a member of a wider interprofessional group and should be associated with the willingness to get to know other professions” (Reinders & Krijnen, 2023, p. 3). Reinders (2018) *and Reinders et al. (*2018*) provided no definition.*	
**Commitment to working interprofessionally**	Khalili et al. (2013) *and* Khalili et al. (2019) Interprofessional commitments	Khalili et al. (2013) and Khalili et al. (2019) provided no definition.	Commitment to an interprofessional way of working, indicated by the enactment of and positive feelings toward continuing interprofessional collaboration
	Reinders (2018), *Reinders and Krijnen (2023), and* Reinders et al. (2018) 1. Interprofessional identity 2. Interprofessional commitment	1. Interprofessional identity = “the degree to which an individual feels a part of and is committed to a group or social category consisting of different professions that pursue joint goals” (Reinders & Krijnen, 2023, p. 2). 2. Interprofessional commitment = “related to positive feelings towards long-term collaboration with other professions and should determine the degree of effort to which an individual wants to collaborate with members of other professions” (Reinders & Krijnen, 2023, p. 3). Reinders (2018) *and Reinders et al. (*2018*) provided no definition.*	
	Cantaert et al. (2022) Commitment to an interprofessional group or community	Commitment = “self-reflective beliefs about someone’s relationship with social groups or adoption of roles” (Cantaert et al., 2022, p. 10).	
**Values, attitudes, beliefs, and ethics related to interprofessional collaboration**	Wood et al. (2022) 1. Interprofessional values 2. Interprofessional beliefs 3. Interprofessional attitudes	1–3. Wood et al. (2022) provided no definition.	Interprofessional values, attitudes, beliefs, and ethics related to interprofessional collaboration, as indicated by, for example, (a) valuing interprofessional collaboration, (b) respecting the culture, values, roles and responsibilities, and expertise of other professions, (c) awareness about one’s own beliefs and stereotypes that might hinder collaboration, and (d) openness towards collaborative strategies that go beyond professional boundaries
	Thistlethwaite et al. (2016), *based on the definition by the* *Interprofessional Education Collaborative Expert Panel* (2011) Values and ethics	*Definition of IPEC 2011 competency domain* Values and ethics = “work with individuals of other professions to maintain a climate of mutual respect and shared values” *(Interprofessional Education Collaborative Expert Panel*, 2011, *p. 19)*.	
	*Hammick et al. (2009), as cited in Thistlethwaite et al. (2016) and* Floyd and Morrison (2014) Conducting oneself the right way during performance^b^	Conducting oneself the right way = “conducting oneself in the right way during performance, including appropriate attitudes and values” *(Hammick et al., 2009, as cited in Thistlethwaite et al.*, 2016, *p. 140)*. Conducting oneself the right way = “doing the task with the appropriate attitudes, and having suitable values and beliefs about what we are doing” *(Hammick et al., 2009, as cited in Floyd & Morrison,* 2014, *p. 8)*.	
	Khalili et al. (2013) *and* Khalili et al. (2019) 1. Interprofessional beliefs 2. Interprofessional values	1–2. Khalili et al. (2013) and Khalili et al. (2019) provided no definition.	
	Reinders (2018), *Reinders and Krijnen (2023), and* Reinders et al. (2018) Interprofessional beliefs	Interprofessional beliefs = “related to goal-related perceptions towards IPC and should lead to a display of behaviours congruent with these identity beliefs” *(Reinders & Krijnen,* 2023, *p.3)*. Reinders (2018) *and Reinders et al. (*2018*) provided no definition.*	
	Cantaert et al. (2022) 1. Core values that professionals hold 2. Awareness of preconceived assumptions 3. Openness to professional diversity 4. Perceived self-efficacy in their capabilities 5. Commitment to an interprofessional group or community	1. Core values = “basic convictions that guide professionals to enact collaborative behaviour across interprofessional situations” *(Cantaert et al.,* 2022, *p. 8)*. 2. Awareness = “awareness about beliefs and stereotypes that might hinder collaboration” *(Cantaert et al.,* 2022, *p. 9)*. 3. Openness = “professionals are not bound by their traditional boundaries of profession-centric thinking, but instead have developed diversity beliefs that counter unfavourable categorization and allow a broader orientation on client-centred collaborative care” *(Cantaert et al.,* 2022, *pp. 9–10)*. 4. Self-efficacy = “beliefs concerned with judgements of how well one can execute courses of action required to deal with prospective situations, including those in interprofessional situations” *(Cantaert et al.,* 2022, *p. 10)*. 5. Commitment = “self-reflective beliefs about someone’s relationship with social groups or adoption of roles” *(Cantaert et al.,* 2022, *p. 10)*.	
**Knowledge and understanding of roles, responsibilities and expertise**	Wood et al. (2022) Interprofessional knowledge	Wood et al. (2022) provided no definition.	Knowledge and understanding of one’s own and other professions’ roles and responsibilities, and expertise
	Thistlethwaite et al. (2016), *based on the definition by the* *Interprofessional Education Collaborative Expert Panel* (2011) Understanding roles and responsibilities of other healthcare professionals	*Definition of IPEC 2011 competency domain* Roles and responsibilities = “use the knowledge of one’s own role and those of other professions to appropriately assess and address the healthcare needs of the patients and populations served” *(Interprofessional Education Collaborative Expert Panel,* 2011), *p. 21)*.	
	*Hammick et al. (2009), as cited in Thistlethwaite et al. (2016) and* Floyd and Morrison (2014) Knowing what to do^b^	Knowing what to do = “thinking about what action is needed and why” *(Hammick et al., 2009, as cited in Thistlethwaite et al.,* 2016, *p. 140)*. Knowing what to do = “knowing the right thing to do” *(Hammick et al., 2009, as cited in Floyd & Morrison,* 2014, *p. 8)*.	
	Cantaert et al. (2022) Awareness of preconceived assumptions	Awareness = “awareness about beliefs and stereotypes that might hinder collaboration” *(Cantaert et al.,* 2022, *p. 9)*.	
**Skills in interprofessional collaboration**	Wood et al. (2022) Interprofessional skills	Wood et al. (2022) provided no definition.	Interprofessional collaborative skills, such as (a) effective and clear communication of role boundaries, (b) integration of different expertise and experiences, (c) engagement of diverse professionals and resources that complement one’s own professional expertise, and (d) reflection on individual and team performance
	Thistlethwaite et al. (2016), *based on the definition by the* *Interprofessional Education Collaborative Expert Panel* (2011) 1. Understanding roles and responsibilities of other healthcare professionals 2. Interprofessional communication 3. Teamwork and collaborative practice	*Definition of IPEC 2011 competency domain* 1. Roles and responsibilities = “use the knowledge of one’s own role and those of other professions to appropriately assess and address the healthcare needs of the patients and populations served” *(Interprofessional Education Collaborative Expert Panel,* 2011, *p. 21)*. 2. Interprofessional communication = “communicate with patients, families, communities, and other health professionals in a responsive and responsible manner that supports a team approach to the maintenance of health and the treatment of disease” *(Interprofessional Education Collaborative Expert Panel,* 2011, *p. 23)*. 3. Teams and teamwork = “apply relationship-building values and the principles of team dynamics to perform effectively in different team roles to plan and deliver patient-/population centred care that is safe, timely, efficient, effective and equitable” *(Interprofessional Education Collaborative Expert Panel,* 2011, *p. 25)*.	
	*Hammick et al. (2009), as cited in Thistlethwaite et al. (*2016*) and* Floyd and Morrison (2014) Having the skills to do what should be done^b^	Having the skills = “being competent and practicing correctly” *(Hammick et al., 2009, as cited in Thistlethwaite et al.,* 2016, *p. 140)*. Having the skills = “being competent and capable of behaving and doing things correctly” *(Hammick et al., 2009, as cited in Floyd & Morrison,* 2014, *p. 8)*.	
	Khalili et al. (2013) *&* Khalili et al. (2019) Interprofessional behaviours	Khalili et al. (2013) and Khalili et al. (2019) provided no definition.	

^a^For examples (if detailed), please see the definitions provided in Online Resource 1

^b^The original book was not available to the authors, so the definitions are taken from other authors citing the book

### Theories explaining interprofessional identity formation

We identified five theories commonly used to explain IPIF: social identity theory, intergroup contact theory, landscapes of practice (LoP), boundary crossing and role identity theory. This section describes the characteristics of the corpus informing these findings and outlines each theory’s relevance to IPIF. The subsequent section provides a thematic overview based on reflexive thematic analysis of how these theories suggest that education may foster IPIF.

#### Characteristics of the literature

The five most commonly used theories were identified from a corpus of 25 works consisting of 23 journal articles and 2 book chapters (see Online Resource 2). Most of the corpus originated from journals in the health professions and health professions education domain, except three articles. The aims of the research represented in our corpus varied greatly, for example, from increasing conceptual understanding of IPI (*n* = 5) to assessing the effects of IPE (*n* = 3), to describing a new framework (*n* = 2), to exploring IPIF in specific settings (*n* = 5).

The study populations in the empirical works in our corpus (*n* = 16) were primarily students or professionals, originating from varying professions and educational programmes in healthcare. None encompassed professionals or students from other disciplines. Works that were not empirical focused on an analysis of organisational sectors (*n* = 5), such as health and social care and (medical/health professions/interprofessional) education, or did not specify a focus (*n* = 2).

#### The five theories most commonly used to explain interprofessional identity formation

An overview of each of the five theories’ origin, central premise, relation to IPC and the mechanisms relevant to IPIF is provided in Table 2. This overview is based on works commonly cited to explain the theories in general and their relation to IPC.

**Table 2:** The origin, central premise, relation to IPC and the mechanisms we consider relevant to interprofessional identity formation for each of the five theories most commonly used to explain interprofessional identity formation

Theory	Origin	Central premise and relation to interprofessional collaboration (IPC)	**Mechanisms relevant to** *interprofessional identity formation*	**Sources**^1^
**Social identity theory**	Social psychology	*Central premise* Individuals categorise themselves and others into social groups, and derive their self-concept and identity from these memberships. ** In-groups** are groups an individual identifies with; the **out-group** is a group that is not their own. *Relation to IPC* This theory explains how learners^2^ identify with their own professional groups and perceive and interact with other professional groups, shaping intergroup dynamics such as rivalry, stereotyping, discrimination and status differences. Intergroup differentiation can hinder IPC in teams when in-group members engage primarily with each other, creating social distance from those perceived to be the out-group.	***Intergroup differentiation****:* During intergroup interactions, in-groups emphasise internal similarities and out-group differences. In-groups reinforce their group identity and self-esteem by favouring themselves over out-groups, stereotyping and unfavourable comparisons of attributes, leading to bias and prejudices.	Haslam et al. (2000), Reinders and Pesut (2022), Tajfel (1978), Tajfel et al. (1971), Tajfel and Turner (1979, 1986),Thistlethwaite et al. (2016), and Turner (2010, 1982).
**Intergroup contact theory**	Social psychology, specifically Allport’s (1954) contact hypothesis	*Central premise* Positive interactions between individuals from different groups reduce prejudice and improve intergroup relations. Four interrelated cognitive processes enhance **intergroup contact** and relationships. *Relation to IPC* Intergroup contact in an open and trusting environment can result in the formation of a unified group, such as is ideal in an interprofessional team.	***Processes enhancing intergroup contact:*** • Learning about out-groups: understanding them to challenge stereotypes. • Changing behaviour: opening up to positive interactions with out-group members, fostering more inclusive attitudes, especially when the contact is repeated. • Building affective ties: continued positive intergroup contact results in affective ties and reduces intergroup anxiety. However, negative intergroup contact experiences can increase intergroup anxiety. • In-group reappraisal: re-evaluating perceptions of in- and out-groups, reducing in-group favouritism and out-group bias and improving intergroup relations.	Allport (1954), and Pettigrew (1998).
**Landscapes of practice (LoPs)**	Sociocultural learning theory, specifically communities of practice (CoPs)	*Central premise* Learning, i.e. knowledge acquisition and identity formation, occurs across CoPs within a broader **landscape of interconnected practices**. Identity formation is dynamic and complex. A **CoP** is a group of individuals united by a shared interest who improve their proficiency through regular interaction. CoPs focus on specific knowledge domains, separated by boundaries. A professional’s **body of knowledge** spans multiple CoPs, requiring learners to navigate this LoP by crossing boundaries between CoPs to integrate diverse perspectives and practices. *Relation to IPC* The boundaries between practices in a LoP can create both challenges to and opportunities for IPC when learners navigate their area of expertise while attempting to integrate the knowledge of others. The sharing of knowledge and practices between learners leads to additions to the LoP and fosters a mutual learning process. The LoP also includes the broader social, organisational and contextual contexts that shape professional practices. These contexts often influence power dynamics, roles and responsibilities within teams and therefore IPC.	*Brokers and boundary objects facilitate connections between CoPs:* Brokers are individuals at the boundaries who help exchange practices, termed “brokering”; boundary objects, such as documents and artefacts, serve the same function. *Modes of identification influencing identity formation:* • Engagement requires active involvement in activities of the LoP; • Imagination involves envisioning one’s role within the LoP, which can be stereotypical and inaccurate; • Alignment involves coordinating actions to fit the LoP, a two-way process distinct from mere compliance. All modes can result in identification or disidentification and are most effective in ensuring identification when they are in balance. *Achieving knowledgeability*: Instead of needing to master all practices in a LoP, individuals can achieve knowledgeability – an awareness of other practices, their relevance, and their location in the broader landscape. Knowledgeable learners engage in boundary crossing and question the relevance of their practice to others, without necessarily joining the other CoP.	Akkerman et al. (2008), Buckley et al. (2019), Törnqvist (2023), Wenger (1998), and Wenger-Trayner and Wenger-Trayner (2015).
**Boundary crossing**	Educational theory, organisational theory, and sociology	*Central premise* Learning and innovation occur at the boundaries between practices. A **boundary** is “a sociocultural difference leading to discontinuity in action or interaction” (Akkerman & Bakker, 2011, p. 133). ** Boundary crossing** presents learning opportunities by exposing individuals to diverse perspectives, practices, and knowledge systems, fostering new understandings and innovative solutions. Four boundary crossing learning mechanisms maintain interaction across boundaries while preserving the boundaries’ unique characteristics. *Relation to IPC* A professional boundary exists where one scope of practice ends and another begins. IPC requires effective and efficient boundary crossing, enabled by its learning mechanisms.	*Boundary objects facilitate cross-boundary communication:* Boundary objects, such as online collaborative platforms and conceptual models, facilitate communication across boundaries by maintaining recognisability across social contexts. *Boundary crossing learning mechanisms:* These mechanisms help individuals negotiate identities and roles in different contexts, fostering personal and professional growth. • Identification involves understanding the expertise and perspectives of the various practices and their interconnectedness; • Coordination involves creating cooperative and routine exchanges to enhance collaboration between practices; • Reflection encourages learners to broaden their perspectives by considering others’ viewpoints; • Transformation involves developing (new) practices through integrating diverse expertise and perspectives.	Akkerman and Bakker (2011), Fortuin et al. (2023), Star and Griesemer (1989), and Thistlethwaite et al. (2016).
**Role identity theory**	Sociology and social psychology	*Central premise* Individuals experientially construct their identities through enacting social roles, enabling self-verification and categorisation by others. A **role identity** is a self-view concerning a specific role, shaped by perceived feedback on role enactment and the associated self-image. This internalised set of role expectations gives a sense of self and guides behaviour. Roles are hierarchically arranged by perceived importance, known as **role salience**. *Relation to IPC* Role identities influence learners’ perception of their role and responsibilities within an interprofessional team. A mismatch between the situational demands and salient role identities can hinder IPC when learners refrain from the expected IPC behaviours. Similarly, recognition and validation of a role identity can foster IPC behaviours.	*Role salience determines behaviour:* Higher role salience makes a role more central to the sense of self and leads to behaviour aligned with role expectations. Role identities are renegotiated based on positive, negative or neutral support received during enactment. In principle, learners strive to maximise support, especially for highly salient and/or threatened role identities. Highly salient role identities are more vulnerable to identity-threatening stressors and benefit more from identity-enhancing events than less salient ones. *Matching situational demands and role identities:* Role salience can fluctuate due to daily demands or unique events. A match between situational demands and expected role behaviours facilitates corresponding behaviours, while mismatches can lead to identity threat and distress, prompting learners to protect their identity by refraining from expected behaviours.	Burke (1991), Burke and Tully (1977), Farmer et al. (2003), Markus and Wurf (1987), Mausz et al. (2022), Siebert et al. (1999), Thoits (1991, 2012), and Thompson et al. (2022).

^1^Sources are presented in alphabetical order; no meaning can be derived from the order of presentation.

^2^Although all theories vary in terminology for the individual undergoing identity formation (e.g., individual, learner), we used “learner” to align with our focus on guidelines for educational design for undergraduate education, where the individual who is studying is often referred to as a “learner”.

### How interprofessional identities are fostered as seen through the lens of the five theories

In this section, we applied the five theories to determine how to foster each of the five IPI themes identified in our literature review (see also Table 1). By connecting these themes with the mechanisms of the theories we considered relevant to IPIF, we identified how to enable the development of the aspects addressed in the five themes according to the theories. Table 3 provides an overview of which mechanisms of each theory foster each theme characterising IPI. Next, we detail how each IPI theme can be enabled through the integration of these mechanisms.

**Table 3:** Mechanisms enabling the five themes characterising interprofessional identity according to the five theories most commonly used to explain interprofessional identity formation.

Theory	Mechanisms enabling the formation of…				
	a sense of belonging to an interprofessional team	a commitment to working interprofessionally	values, attitudes, beliefs, and ethics related to interprofessional collaboration	knowledge and understanding of roles, responsibilities, and expertise	interprofessional collaboration skills
**Social identity theory**	• Emphasise similarities • Challenge stereotypes and biases	• Emphasise similarities • Challenge stereotypes and biases	• Emphasise similarities to enable positive attitudes • Challenge stereotypes and biases	• Challenge stereotypes and biases	No relevant mechanisms described.
**Intergroup contact theory**	• Learn about out-groups • Challenge stereotypes and negative views • Enable empathetic connections and repeated positive experiences	• Enable repeated positive intergroup contact	• Learn about out-groups • Counter stereotypes and negative views • Enable empathetic connections and repeated positive experiences of intergroup contact	• Enable awareness of stereotypes and biases • Learn about out-groups’ roles and responsibilities, and expertise • Challenge stereotypes and biases	No relevant mechanisms described.
**Landscapes of practice**	• “Engagement”: Actively engage in IPC activities and interactions • “Imagination”: Imagine what it would be like to belong to the interprofessional group • “Alignment”: Coordinate practices to ensure effective IPC • Balance engagement, imagination and alignment • Challenge stereotypes and bias within the imagination process	• Enable identification with the interprofessional LoP • Enable the development of knowledgeability • Enable interaction with other learners to experience different practices • Challenge stereotypes and bias	• Experience the interprofessional community’s shared goals, beliefs, values and behaviours • Facilitate accepting of core attitudes and beliefs • Balance engagement, imagination and alignment to ensure identification with the community’s beliefs and values	• Enable the development of knowledgeability • Enable interaction with other learners to experience different practices • Ensure that learning occurs at the boundaries of the practices involved, focussing on the relevance of one practice to another • Make boundaries explicit • See boundaries as learning opportunities • Use boundary objects to enable cross-boundary communication	• Enable the development of knowledgeability • Enable interaction with other learners to experience different practices • Use knowledgeable individuals as role models • “Alignment”: Coordinate practices to ensure effective IPC • Enable experience of boundaries to become competent at boundary crossing throughout an LoP
**Boundary crossing**	No relevant mechanisms described.	• Enable repeated positive boundary crossing experiences	• “Reflection”: Become aware of one’s own practice through one’s own and others’ eyes	• Enable experience of boundaries to see where one scope of practice ends and another begins • “Identification”: Learn about the various types of expertise of the practices in relation to another • Make boundaries explicit • See boundaries as learning opportunities • Use boundary objects to enable cross-boundary communication	• Enable boundary crossing competence • Use competent brokers as role models • “Coordination”: Stimulate learners to coordinate cross-boundary collaboration • “Transformation”: Collaborate to (co)develop a (new) practice (or product)
**Role identity theory**	• Give positive support during the enactment of the interprofessional role identity • Explicate new behaviours and role expectations for professional and interprofessional role identity	• Ensure corresponding situational demands and positive support during enactment of the interprofessional role identity • Increase role identity salience to ensure engagement in interprofessional behaviour	• Ensure positive support during interprofessional role enactment • Increase role identity salience to increase valuing of IPC	• Learn about each other’s role identities and the concurrent internalised expected behaviour	• Make the expected behaviour and related skills for the interprofessional role identity explicit

#### Sense of belonging to an interprofessional team

With a sense of belonging to an interprofessional team, we refer to a feeling of belonging, where individuals view themselves as member of an interprofessional team consisting of individuals from different professions that pursue joint goals.

To enable this sense of belonging, it is important that learners view the interprofessional group as their in-group. Accordingly, learners should learn about the out-groups other learners belong to, and similarities between learners should be emphasised, differences made explicit and stereotypes and biases challenged. Enabling empathetic connections and repeated positive experiences is key during intergroup contact. To ensure identification with the team, the three modes of identification from LoP theory – engagement, imagination and alignment – should be in balance. Lastly, the expectations, for example, concerning behaviours, for both the interprofessional and professional role identity within an interprofessional team should be made explicit. Since misalignment between the situational demands and role identities can lead to identity threat and consequent non-expression of the identity, this could potentially limit professional contributions to IPC and thus IPC’s overall success. Positive support during the enactment of the role of “interprofessional team member” makes the role identity more salient and thus more central to the sense of self, ensuring a stronger sense of belonging.

#### Commitment to working interprofessionally

A commitment to an interprofessional way of working involves enacting and showing positive feelings towards continuing IPC. Hence, the commitment is not temporary, as when an interprofessional team exists during a limited time period, but is instead present continuously and can be triggered by the formation of a new interprofessional team.

A commitment to working interprofessionally requires a positive appraisal of the interprofessional team, enabled by emphasising similarities and challenging stereotypes within repeated positive intergroup contact. A commitment further requires knowledgeability and identification with the interprofessional LoP, which are enabled by learners experiencing different practices through interacting with each other across boundaries. Repeated positive boundary crossing experiences ensure increased commitment. Furthermore, positive support upon enactment of the role and alignment between the situational demands and the role identity increases interprofessional role identity salience. Consequently, learners are more likely to engage in interprofessional behaviours, showing greater commitment towards working interprofessionally.

#### Values, attitudes, beliefs, and ethics related to interprofessional collaboration

This theme focuses on the learner having interprofessional values, attitudes, beliefs and ethics that reflect a positive appraisal of IPC and the interprofessional team. Examples include valuing IPC, respecting other professions, and awareness of beliefs that might hinder IPC.

To enable constructive interprofessional values, attitudes, beliefs, and ethics, it is again important to emphasise similarities between learners and challenge stereotypes and biases. This is fostered by learning about out-groups within the interprofessional team to open up to potential positive IPC experiences and changes in attitudes, further stimulated by empathetic connections between team members and repeated positive experiences of interaction. Through experiencing the interprofessional community’s shared goals, beliefs, values, and behaviours, acceptance of the community’s core attitudes and beliefs, which is necessary to become a community member, is facilitated. Furthermore, identification with the community’s beliefs and values is enhanced by balancing engagement, imagination and alignment, the three modes of identification of LoP theory. According to boundary crossing theory, when learners engage in reflection, they become aware of their practice through their own and others’ eyes, leading to potential changes in values, attitudes, beliefs and ethics. Lastly, the valuing of IPC can be fostered through increased role identity salience, which is enabled by ensuring positive support during interprofessional role enactment.

#### Knowledge and understanding of roles and responsibilities, and expertise

This theme refers to learners’ IPC awareness, knowing and understanding of not only their profession’s roles and responsibilities, skills and expertise but also those of the other team members.

Knowledge and understanding are enabled by learning about out-groups’ roles and responsibilities and their expertise, along with challenging stereotypes and biases, specifically regarding roles and responsibilities. Interaction with other learners to experience different practices can foster knowledgeability in learners, of which knowledge and understanding of the relevance of one practice for another is a big component. Given that learners likely originate from different practices, learning should occur at the boundaries of these practices, allowing learners to see where one scope of practice ends and another begins, known as “identification” in boundary crossing theory. Lastly, making boundaries explicit, considering them learning opportunities, and stimulating learners to use boundary objects to enable cross-boundary communication results in increased knowledge and understanding.

#### Interprofessional collaboration skills

IPC skills refer to interprofessional collaborative skills or competencies, including, but not limited to learners being competent at effectively and clearly communicating role boundaries, engaging with professionals and resources that complement their professional expertise, and reflecting on individual and team performance.

Learners should become knowledgeable by interacting with other learners to experience different practices, which is enhanced when knowledgeable individuals engage in role modelling. Learners coordinate practices to ensure effective IPC through engaging in “alignment”. Along with being knowledgeable, learners should become competent at boundary crossing throughout an LoP by repeatedly experiencing and engaging with boundaries through “coordination” and “transformation”. Learners should be stimulated to collaborate across borders to develop or codevelop a (new) practice or product, in which competent brokers must be used as role models. To ensure the development of constructive interprofessional skills, the expected behaviour and related skills for the interprofessional role identity should be made explicit.

#### Prospective outcomes of interprofessional identity formation according to the five theories

Combining the mechanisms proposed by each of the five theories should result in IPIF. Nonetheless, each theory provided different prospective outcomes of this process, as shown in Table 4.

**Table 4:** The prospective outcomes of interprofessional identity formation according to the five theories.

Theory	Prospective outcome of interprofessional identity formation
Social identity theory	Learners **regard the interprofessional group as an in-group** by **socialising into this group**. All individual group members are regarded as in-group instead of out-group members. In this process, intergroup differentiation is prevented by **emphasising similarities among group members**.
Intergroup contact theory	Learners **regard the interprofessional group as an in-group** as a result of **positive intergroup contact in an open and trusting environment**. Here, learners **learn about each other**’s (out-group) perspectives, experiences and characteristics, eventually resulting in the formation and positive appraisal of a new interprofessional group.
Landscapes of practice	Learners **view themselves as a member of an interprofessional landscape of practice** (LoP). Additionally, learners **become knowledgeable** about the interprofessional LoP. Learners negotiate their multiple identities during boundary crossing; thus, **learning takes place at the boundaries** of the practices learners originate from.
Boundary crossing	Learners **become competent in boundary crossing** as a result of **positive boundary crossing experiences**.
Role identity theory	Learners **develop** **an interprofessional role identity**, ensuring an interprofessional sense of self and corresponding behaviours. This role identity is **highly salient in interprofessional environments** and co-exists alongside the role identities related to learners’ (future) profession.

### Towards guidelines for educational design fostering interprofessional identity formation in interprofessional education

**Fig. 1:**
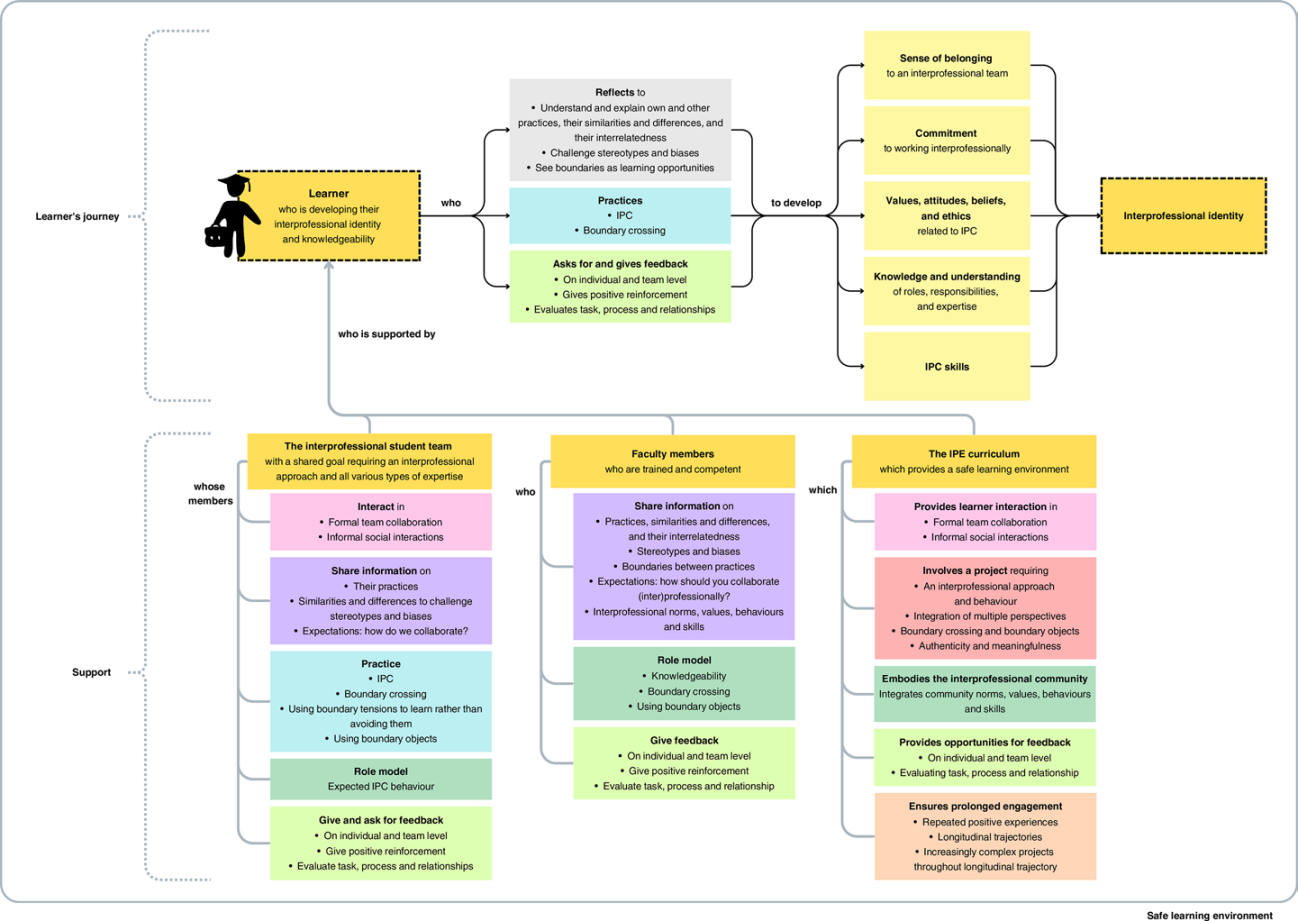
The educational design framework for fostering interprofessional identity formation (ED-IPIF). The framework shows a learner in IPE, who is supported by the interprofessional team, faculty members and the IPE curriculum. Similar colours indicate thematic overlap between building blocks, except for the yellow colour identifying the learner, team, faculty, curriculum and interprofessional identity. For clarity, the ED-IPIF is shown as a linear pathway; in practice, reflection, practice, and feedback interact in cyclical and iterative ways. Abbreviations: IPC = interprofessional collaboration; IPE = interprofessional education

In this section, we describe our framework for educational design, called the Educational Design framework for fostering Interprofessional Identity Formation (ED-IPIF; Fig. 1). The ED-IPIF is based on the mechanisms relevant to IPIF of the five theories discussed in this paper.

When considering IPE to be the main educational approach through which IPIs can be formed, there are four areas to be addressed: the learners, their interprofessional student teams, the faculty members and the IPE curriculum. The ED-IPIF in Fig. 1 visualises 1) the learner’s journey towards developing an IPI and knowledgeability, and 2) the support the learner requires from the student team, faculty and curriculum. In the following, we explain the building blocks of the ED-IPIF. The similar colours in the framework indicate thematic overlap between blocks, except for the yellow colour identifying five key constructs: the learner, interprofessional team, faculty, IPE curriculum and IPI.

The learner’s journey includes four building blocks. The first building block is a learner who is aiming for an IPI and interprofessional knowledgeability. While knowledgeability enables successful collaboration across professional boundaries (Wenger-Trayner & Wenger-Trayner, 2015), IPI provides the necessary foundation and direction for IPC competencies and promotes interprofessional collaborative behaviours (Ganotice, 2023; Flood et al., 2019). Second, the learner engages in a learning process involving reflection, practice, and asking and giving feedback. Third, the learner develops the five IPI aspects identified in our themes, finally resulting in IPIF. Although the ED-IPIF suggests a linear pathway, we realise that identity formation is not linear (see also Polansky et al., 2023) and that reflection, practice and feedback can influence each other and take place as an iterative cyclical process. However, for purposes of clarity, the framework is presented as simply linear.

In the ED-IPIF, support takes three different shapes. First, the individual learner is part of an interprofessional team that should be built up of learners having different types of expertise. The team should have a shared goal, such as a project to complete, that requires all of these types of expertise, to ensure interdependence. Together, the team members should engage in boundary crossing, using boundary objects to stimulate cross-boundary communication. The team and its members provide opportunities for interaction and practice, showcasing expected behaviours, and feedback for the individual learner. While developing an IPI and knowledgeability, learners may participate in multiple teams with varying compositions. While we acknowledge that IPIF can emerge from various interactive experiences, including outside of teams (see, e.g., Dolan & Nowell, 2023), this paper focuses on collaboration within teams and excludes other experiences from the ED-IPIF. Second, support is offered by the faculty involved. Faculty members should be trained and competent and represent an interprofessional set of types of expertise. Faculty members function as a source of information and feedback for the learner, and as a role model for expected behaviour within the interprofessional community. The importance of faculty as interprofessional role models for students’ interprofessional development is underscored by, for example, Price et al. (2021) and Cimino et al. (2022).

Third, support is offered through the IPE curriculum, which supports learners by being a safe learning environment and fostering continuous development of IPI and knowledgeability through longitudinal trajectories. It offers the learner opportunities for interaction and feedback through the interprofessional project assignment, with relevant stakeholders, such as patients or policy makers, in authentic and meaningful contexts (see also Maddock et al., 2023). These projects should progressively increase in complexity, uncertainty, and divergence, challenging learners with increasingly wicked problems throughout the curriculum, as supported by research on students’ boundary crossing learning in response to wicked problems in higher education by Veltman (2024) and Veltman et al. (2019). Lastly, the curriculum design explicitly reflects and showcases the interprofessional community, encompassing both the formal IPE curriculum and the hidden curriculum. The hidden curriculum includes the implicit cultural norms, values and expectations conveyed through both formal and informal educational practices (Hafferty & Franks, 1994). This includes spontaneous interpersonal interactions, role modelling, and implicit messages (Gaufberg & Hafferty, 2021), about how professions interact, collaborate, and communicate. Ensuring alignment between the formal and hidden curricula in reflecting the interprofessional community is crucial. Together, the team, faculty, and curriculum can and should contribute to creating a psychologically safe learning environment where students feel safe to be themselves, speak up, collaborate or practice without repercussion, such as rejection, from the team (Edmondson, 1999; Maddock et al., 2023). Psychological safety requires mutual respect and trust among team members (Edmondson, 1999). Whereas a lack of psychological safety inhibits learning behaviours, perceived psychological safety enables learning behaviours at the individual and team level, including taking risks, acknowledging mistakes, asking for help and giving and seeking feedback (Aranzamendez et al., 2015; Edmondson, 2019; Newman et al., 2017). Therefore, a psychologically safe learning environment contributes to the learner’s IPIF.

Power imbalances and hierarchies are known to negatively affect psychological safety (see e.g., Edmondson et al., 2016; Appelbaum et al., 2020), as does misalignment between the hidden and formal curriculum (see e.g., Meyer et al., 2025). Although not explicitly addressed in the five theories underpinning the ED-IPIF, both power and hierarchy and the hidden curriculum are highly influenced by role modelling, which the ED-IPIF does include. For example, if faculty reinforce power imbalances or stereotypes through their behaviour, learners may adopt these behaviours (Appelbaum et al., 2020). Similarly, the hidden curriculum is largely conveyed through role modelling (Meyer et al., 2025). This creates opportunities to model norms, values and expected behaviours of the interprofessional community through the actions of the student team, faculty and IPE curriculum.

When considering all the types of support provided to the learner by the interprofessional team, faculty and IPE curriculum, thematic overlap is present, as shown by similar colours in the ED-IPIF. Although the way the support is shaped differs, identifiable overall themes include fostering learner interaction, sharing information, practising together, role modelling and embodying the interprofessional community, and giving and asking for feedback. These, together with the presence of a safe learning environment, are key in supporting the learner’s journey towards IPIF.

## Discussion

To better prepare students in IPE at an undergraduate level for a future in which IPC is required to address the wicked problems they will be confronted with, it is essential to go beyond developing interprofessional collaborative competencies by focusing on fostering IPI. Despite recognition of its importance, the translation of IPI to educational practice interventions has been limited by varying definitions and conceptualisations, and the absence of a framework on how to foster its formation. Therefore, this critical review identified (1) the main characteristics of an IPI and (2) guidelines for educational fostering IPIF.

Our literature review highlighted key themes characterising IPI: 1) a sense of belonging to an interprofessional team; (2) a commitment to working interprofessionally; (3) values, attitudes, beliefs, and ethics related to IPC; (4) knowledge and understanding of roles, responsibilities and expertise in IPC; and (5) IPC skills. Moreover, our review identified five theories commonly used to explain IPIF. Using reflexive analysis, we developed the Educational Design framework for fostering Interprofessional Identity Formation (ED-IPIF) to support this process. Our guidelines for educational design aim to support learners in developing their IPI and knowledgeability through reflection, practice, and feedback, with support from their team, faculty, and curriculum. Teams offer interaction, practice and feedback opportunities, faculty provide guidance and act as role models, and the curriculum ensures prolonged engagement in interprofessional projects with varying degrees of wickedness and relevant stakeholders, such as patients. Together, the team, faculty and curriculum should create a psychologically safe learning environment essential for the learner’s journey towards IPIF and knowledgeability.

Our critical review and ED-IPIF together address several needs related to the lack of conceptual clarity on IPI and of a framework for how to foster IPIF. First, while current literature suggests simply implementing IPE that fosters IPIF, our review shows that designing such education is challenging. IPE fostering IPIF must simultaneously address multiple interrelated areas subject to complex interaction. The types of support provided by aspects of the learner’s environment cannot be considered or implemented on their own. Instead, IPE fostering IPIF requires an integrated approach.

Second, our review shows that while conceptualisations of IPI vary, there is considerable overlap that allows them to be synthesised into five overarching themes. Although the field of IPIF remains fragmented with multiple definitions, conceptualisations and measurement approaches (Tong et al., 2020), these findings contribute to the development of a more unified theoretical foundation for IPI research.

Third, the ED-IPIF and our guidelines for educational design have a strong theoretical foundation, in response to Paradis and Whitehead’s (2018) and Hean et al.’s (2018) call for more theory-based IPE. We grounded our guidelines in the mechanisms of the five theories most commonly used to explain IPI, incorporating a broader theoretical base than most studies. Often, explanations of IPIF rely solely on social identity theory and intergroup contact theory (Paradis & Whitehead, 2018; Tong et al., 2020), but framing IPI purely in terms of group membership may not adequately capture the barriers and enablers associated with IPIF (Tong et al., 2020). For example, IPE designed based on only intergroup contact theory, assumes that simply bringing different groups together reduces prejudice and fosters positive group relationships; yet, forced interaction can reinforce stereotypes, especially if existing power dynamics are ignored (Paradis & Whitehead, 2015). IPE design therefore requires a broader theoretical basis. Although theories such as LoP and boundary crossing are gaining traction in explaining IPIF, they still lack specific guidance for IPE design.

Fourth, we move beyond existing theories on IPIF (Cantaert et al., 2022; Khalili et al., 2013; Reinders, 2018) by explicitly detailing the educational elements necessary to foster IPIF, moving from theoretical mechanisms towards concrete guidelines for educational design. Lastly, the ED-IPIF uniquely integrates IPIF with IPC competencies and provides a much-needed integration of principles, concepts and practices in the literature on IPI and IPIF in IPE.

### Recommendations for future research

Our recommendations for future research are twofold: addressing the gaps in the literature and empirically testing the ED-IPIF while developing robust measurement instruments to do so.

During our review process, we identified several literature gaps. First, the five theories commonly used to explain IPIF overlook power and hierarchy, despite their well-documented negative effects on IPC (Baker et al., 2011; Olson & Brosnan, 2017; Paradis & Whitehead, 2015; Polansky et al., 2023). Although IPE often implicitly or explicitly aims to address power and conflict, limited research explores how IPE tackles these issues (Paradis & Whitehead, 2015, 2018). Existing studies on power in interprofessional settings have, for example, focused on how managers can mitigate power imbalances (Okpala, 2020), how power distance, psychological safety and team cohesion influence team effectiveness (Appelbaum et al., 2020), and how perceived roles and power dynamics shape healthcare trainee interactions (Gergerich et al., 2019). While power and hierarchy did not emerge explicitly in our literature review, they are implicitly addressed in the ED-IPIF through role modelling performed by the student teams, faculty, and curriculum, and by efforts to counter stereotypes and biases. Still, we believe this does not capture the full complexity of power and hierarchy in IPE. Therefore, we agree with Paradis and Whitehead (2018) that future research should develop theory-based approaches that explicitly address tackling power and hierarchy within IPE to better foster IPIF and IPC.

Next, the five commonly used theories also fail to consider the wider context in which IPE is situated, such as the programme, faculty, educational institution, and country. Educational environments include both the formal curriculum and all that occurs outside the formal curriculum, known as the hidden curriculum, conveyed through the institution’s structure, language, resource allocation and policy (Gaufberg & Hafferty, 2021). The hidden curriculum of the host institution influences IPE, IPC and IPIF, and is influenced by the broader societal context. Therefore, the institutional systems must recognise the importance of IPE and IPC, and embody the interprofessional community. Although the hidden curriculum did not appear explicitly in our literature review, it is reflected in the ED-IPIF through the role modelling by students, faculty and curriculum of the interprofessional community’s norms, values and expected behaviours. While our guidelines for educational design focus on curricular implementation, sustainable impact requires broader commitment. Therefore, future research could explore how the hidden curriculum shapes IPIF, for example, by comparing the interprofessional norms and values of the curricula across educational institutions.

The last literature gap concerns the lack of a longitudinal perspective on how IPI develops, since the IPE field is characterised by short-term interventions. While the ED-IPIF acknowledges that IPIF is cyclical and dynamic, the specific process remains unclear. Yet, exploring mediating and moderating factors and mechanisms is crucial for our understanding of the link between IPI and IPE-related outcomes (Ganotice, 2023; Paradis & Whitehead, 2018). What also remains unclear is the optimal timing of IPE, as views differ on whether it should be early or later on in the educational program (Paradis & Whitehead, 2018). Therefore, longitudinal studies are needed to determine how IPI forms over time, identify key influencing factors and establish the optimal timing for IPE fostering IPIF. The ED-IPIF can serve as a valuable tool for studying these effects and understanding the mechanisms at play.

As of now, the ED-IPIF provides concrete starting points for educational design, but its descriptive nature necessitates empirical testing. Empirical testing allows for a translation of our guidelines into design principles for education fostering IPIF, which is our ultimate aim. Therefore, the next step is to develop IPE informed by our guidelines and to assess how and whether it effectively fosters IPIF. To do this, we recommend using design-based research, which considers the unique context of each educational program (Dolmans & Tigelaar, 2012; McKenney & Reeves, 2018). A critical part of this research will be operationalising the five themes of IPI to be able to determine whether IPE, for example, leads to a greater sense of belonging. However, the current lack of robust measurement instruments complicates this, as many existing tools suffer from poor construct validity (Tong et al., 2020). Additionally, the successful implementation of IPE is inherently complex, with factors such as funding, faculty development, organisational structures, and staff commitment serving as both barriers and enablers, depending on the context (Lawlis et al., 2014; Paradis & Whitehead, 2018). Therefore, future research should focus on testing the ED-IPIF across diverse educational settings, taking the context and concurrent barriers and enablers into account, while simultaneously developing effective measurement tools for IPI.

### Practical implications for educational design

Our critical review and the ED-IPIF offer valuable insights for educational institutions and designers aiming to design IPE fostering IPIF.

For educational institutions, our review underscores the need to recognise and emphasise the importance of IPI in practice. We recommend that institutions assess how and to what extent their environment and hidden curriculum reflect and support the interprofessional community. Furthermore, in line with Lawlis et al. (2014), we recommend that institutions develop or refine their vision and strategies for IPE, including how IPE is integrated into curricula, resource distribution, and policy development (Lawlis et al., 2014). Since the ideal timing for IPE remains debated (Paradis & Whitehead, 2018), institutions must decide how and when to best support their students in developing IPC competencies and IPIs, prioritising longitudinal trajectories and increasing complexity of problems.

For educational designers, the ED-IPIF offers concrete starting points for course design. It emphasises the importance of focusing on individual learners’ growth in IPI and knowledgeability. Key contributors to this process include the interprofessional team, faculty, and the IPE curriculum, each supporting the individual learner in their own way. The ED-IPIF deliberately does not elaborate on specific teaching and learning activities, as shaping educational design is highly context-specific (Krystallidou et al., 2024). Instead, we aimed to provide an overview of guidelines for educational design that can easily be adapted to specific educational contexts. Consequently, we highly encourage educational designers to tailor the guidelines to their IPE programs and associated stakeholders, such as patients or clients, using the vast number of example activities in the field as inspiration.

### Conclusions

Fostering IPIF in IPE provides opportunities to ensure that health professions students are optimally prepared for IPC to tackle wicked problems in their future field of work. Moving beyond the simple statement that IPIF should be part of IPE, the ED-IPIF offers concrete guidelines for educational practice. Using this framework, we can foster knowledgeable learners with an IPI that is vital to working on wicked problems in an interprofessional or interdisciplinary setting.

## Electronic supplementary material

Below is the link to the electronic supplementary material.

Supplementary Material 1

Supplementary Material 2

## Data Availability

No datasets were generated or analysed during the current study.
