# Supplementary material for: Fostering interprofessional identity formation to support interprofessional collaboration – Identifying guidelines for educational design
Source: Adv Health Sci Educ Theory Pract. 2025 Oct 20;31(3):1023–54. doi: 10.1007/s10459-025-10478-9 (PMC13234016; doi:10.1007/s10459-025-10478-9)
Supplement: Supplementary file 1 — Supplementary Material 1 [file 10459_2025_10478_MOESM1_ESM.pdf]

Online Resource 1: Definitions and/or aspects of interprofessional identity

Article title: Fostering Interprofessional Identity Formation to Support Interprofessional Collaboration – Identifying Guidelines for Educational Design

Journal: Advances in Health Sciences Education

Author list: Annemarie B. Sänger[1], Renée E. Stalmeijer[1], Simon Beausaert[2], Jascha de Nooijer[1]

1. School of Health Professions Education, Faculty of Health, Medicine and Life Sciences, Maastricht University, the Netherlands
2. Educational Research and Development, School of Business and Economics, Maastricht University, the Netherlands

Corresponding author: Annemarie B. Sänger, School of Health Professions Education, Faculty of Health, Medicine and Life Sciences, Maastricht University, the Netherlands.
a.sanger@maastrichtuniversity.nl

This Online Resource provides the definitions and/or aspects of interprofessional identity that resulted from our literature search and were used as input to yield our five themes characterising interprofessional identity.

Table 1. Definitions and/or aspects of interprofessional identity (IPI) sorted alphabetically by first author name

**Article**	**Definition and/or aspects of interprofessional identity**
Cantaert et al. (2022)	Attributes of an interprofessional identity:<br>*Content-related*<br>- Core values that professionals hold = "basic convictions that guide professionals to enact collaborative behaviour across interprofessional situations" *(p. 8)*, e.g., respect.<br>- Awareness of preconceived assumptions = "awareness about beliefs and stereotypes that might hinder collaboration" *(p. 9)*, e.g., valuing of collaboration.<br>- Openness to professional diversity = "professionals are not bound by their traditional boundaries of profession-centric thinking, but instead have developed diversity beliefs that counter unfavourable categorization and allow a broader orientation on client-centred collaborative care" *(pp. 9-10)*, e.g., being positive about shared goal setting.<br>- Perceived self-efficacy in their capabilities = "beliefs concerned with judgements of how well one can execute courses of action required to deal with prospective situations, including those in interprofessional situations" *(p. 10)*, e.g., being confident about sharing leadership.<br>- Commitment to an interprofessional group or community = "self-reflective beliefs about someone's relationship with social groups or adoption of roles" *(p. 10)*, e.g., perceiving a sense of belonging.<br>*Structure-related*<br>- Context-dependency = "fit between the individual and the interprofessional environment, which is an important determinant of the salience of IPI" *(p. 12).*<br>- Development of a team mental model, e.g., sense of working side by side. A team mental model is "a shared mental model of collaborative teamwork, which builds on a mutual commitment of professionals

Article	Definition and/or aspects of interprofessional identity
	working in an interprofessional team" *(p. 12)*; "the mental representations that team members share and use to describe, explain and predict group-level phenomena" *(p. 12).*<br>- Fluid and dynamic development throughout the lifespan of a professional = "the ongoing process through which one integrates their past, present and future self, which proceeds in a nonlinear way following steady and turbulent stages" *(p. 13).*<br>- Interprofessional identity development requires calibration, "through which the cognitive, social and emotional capacities required for the meaning-making of collaborative practice are integrated (e.g., transcending professional boundaries)" *(p. 7)*. […] "A calibration process depicts the learning trajectory when individuals construct step-by-step coherent values and beliefs that fit the required awareness, openness, self-efficacy and commitment that have been defined as the content-related attributes of IPI." *(p. 13).*
Thistlethwaite et al. (2016) and Floyd & Morrison (2014) citing Hammick et al. (2009)	Being interprofessional entails:<br>- Knowing what to do:<br>o "Thinking about what action is needed and why" *(Hammick et al., 2009, as cited in Thistlethwaite et al., 2016, p. 140)*.<br>o "Knowing the right thing to do" *(Hammick et al., 2009, as cited in Floyd & Morrison, 2014, p. 8)*.<br>- Having the skills to do what should be done:<br>o "Being competent and practicing correctly" *(Hammick et al., as cited in Thistlethwaite et al., 2016, p. 140)*.<br>o "Being competent and capable of behaving and doing things correctly" *(Hammick et al., as cited in Floyd & Morrison, 2014, p. 8)*.<br>- Conducting oneself in the right way during performance:<br>o "Conducting oneself in the right way during performance, including appropriate attitudes and values" *(Hammick et al., 2009, as cited in Thistlethwaite et al., 2016, p. 140)*.<br>o "Doing the task with the appropriate attitudes, and having suitable values and beliefs about what we are doing" *(Hammick et al., 2009, as cited in Floyd & Morrison, 2014, p. 8)*.<br><br>Note: The definitions attributed to Hammick et al. (2009) originate from Thistlethwaite et al. (2016) and Floyd and Morrison (2014), as the original book was not available to the authors.
Khalili et al. (2013) and Khalili et al. (2019)	Khalili et al. (2013): Interprofessional identity is part of a dual identity together with professional identity.<br><br>Khalili et al. (2019): Dual identity = "the development of robust sense of belonging to both own profession (in-profession favouritism) and to the interprofessional community (interprofessional favouritism) in which individuals view themselves simultaneously as a member of their own profession and the interprofessional team/community" *(p. 28)*.<br><br>Khalili et al. (2019): Interprofessional socialization = "a process in which individuals develop a dual professional and interprofessional identity (dual identity) through acquisition of both professional and interprofessional beliefs, values, behaviors, and commitments to become 'collaborative

Article	Definition and/or aspects of interprofessional identity
	practice-ready' to practice collaboratively with others to address the quadruple aim" *(p. 26).*<br><br><u>Note:</u> Based on the definition by Khalili et al. (2019), we deduce that the interprofessional identity aspects identified by these author teams entail:<br>- Beliefs<br>- Values<br>- Behaviours<br>- Commitments<br>These aspects are not further defined within the articles of Khalili et al. (2013) and Khalili et al. (2019).
Reinders (2018), Reinders & Krijnen (2023), and Reinders et al. (2018)	Reinders (2018) and Reinders et al. (2018): Interprofessional identity is superordinate to professional identity.<br><br>Reinders & Krijnen (2023): Interprofessional identity = "the degree to which an individual feels a part of and is committed to a group or social category consisting of different professions that pursue joint goals" *(p. 2)*.<br><br>Reinders and Krijnen (2023): Interprofessional identity constitutes:<br>- Interprofessional belonging = "related to social inclusiveness of one's own profession as a member of a wider interprofessional group and should be associated with the willingness to get to know other professions" *(p. 3)*.<br>- Interprofessional commitment = "related to positive feelings towards long-term collaboration with other professions and should determine the degree of effort to which an individual wants to collaborate with members of other professions" *(p. 3)*.<br>- Interprofessional beliefs = "related to goal-related perceptions towards IPC and should lead to a display of behaviours congruent with these identity beliefs" *(p. 3)*, in which IPC is an abbreviation for "interprofessional collaboration".<br><br>Reinders & Krijnen (2023): These dimensions are 'chosen' because a social identity consists of these dimensions (see Tajfel, 1978) and interprofessional identity is considered a social identity.
Thistlethwaite et al. (2016)	The competencies that constitute being interprofessional often include:<br>- Values and ethics<br>- Understanding roles and responsibilities of other healthcare professionals<br>- Interprofessional communication<br>- Teamwork and collaborative practice<br><br>These competencies are based on the four competency domains (CDs) of the IPEC Core Competencies (2011), which were updated in 2016 after this article was published. The general competency statements are divided into specific competencies. The following quotes show the definitions of the CDs within the IPEC Core Competencies (2011):<br>• CD 1: Values and ethics for interprofessional practice = "work with individuals of other professions to maintain a climate of mutual

Article	Definition and/or aspects of interprofessional identity
	respect and shared values" *(p. 19)*, e.g., respecting the cultures, values, roles/responsibilities, and expertise of other professions.<br>• CD 2: Roles and responsibilities = "use the knowledge of one's own role and those of other professions to appropriately assess and address the healthcare needs of the patients and populations served" *(p. 21)*, e.g., recognizing one's limitations in skills, knowledge and abilities; engaging diverse healthcare professionals who complement one's own professional expertise, as well as their associated resources.<br>• CD 3: Interprofessional communication = "communicate with patients, families, communities, and other health professionals in a responsive and responsible manner that supports a team approach to the maintenance of health and the treatment of disease" *(p. 23)*, e.g., choosing effective communication tools and techniques; expressing one's knowledge and opinions to team members involved in patient care with confidence, clarity and respect, working to ensure common understanding of information.<br>• CD 4: Teams and teamwork = "apply relationship-building values and the principles of team dynamics to perform effectively in different team roles to plan and deliver patient-/population centred care that is safe, timely, efficient, effective and equitable" *(p. 25)*, e.g., integrating the knowledge and experience of other professions […] in shared patient-centred problem-solving; applying leadership practices that support collaborative practice and team effectiveness; reflecting on individual and team performance for individual, as well as team, performance improvement.
Tong et al. (2020)	Interprofessional identity = "a robust cognitive, psychological and emotional sense of belonging to an interprofessional community(s), needed to achieve context-dependent goals" *(p. 6)*.<br><br>These aspects are not further defined within the article of Tong et al. (2020).
Wood et al. (2022)	"IP identity may be comprised of IP values, beliefs, attitudes, knowledge, skills, and attributes that drive IP behavior, or IPCP." *(p. 2)*, in which IP is an abbreviation for "interprofessional" and IPCP is an abbreviation for "interprofessional collaborative practice".<br><br>These aspects are not further defined within the article of Wood et al. (2022).
