# Supplementary material for: Fostering interprofessional identity formation to support interprofessional collaboration – Identifying guidelines for educational design
Source: Adv Health Sci Educ Theory Pract. 2025 Oct 20;31(3):1023–54. doi: 10.1007/s10459-025-10478-9 (PMC13234016; doi:10.1007/s10459-025-10478-9)
Supplement: Supplementary file 2 — Supplementary Material 2 [file 10459_2025_10478_MOESM2_ESM.pdf]

Online Resource 2: Characteristics of the works referencing the five theories most commonly used to explain interprofessional identity formation

Article title: Fostering Interprofessional Identity Formation to Support Interprofessional Collaboration – Identifying Guidelines for Educational Design

Journal: Advances in Health Sciences Education

Author list: Annemarie B. Sänger[1], Renée E. Stalmeijer[1], Simon Beausaert[2], Jascha de Nooijer[1]

1. School of Health Professions Education, Faculty of Health, Medicine and Life Sciences, Maastricht University, the Netherlands
2. Educational Research and Development, School of Business and Economics, Maastricht University, the Netherlands

Corresponding author: Annemarie B. Sänger, School of Health Professions Education, Faculty of Health, Medicine and Life Sciences, Maastricht University, the Netherlands.
a.sanger@maastrichtuniversity.nl

This Online Resource provides the characteristics of the works identified in our literature search that reference the five theories most commonly used to explain interprofessional identity formation.

Table 1. Characteristics of works referencing social identity theory, intergroup contact theory, landscapes of practice, boundary crossing and/or role identity theory to explain interprofessional identity (IPI) formation

**Characteristic**	**Journal article (*n* = 23)**	**Book chapter (*n* = 2)**
Aim		
- Assess the effects of an IPE program on…		
○ Interprofessional socialisation process and dual identity development	1	0
○ Students' attitudes and stereotypes concerning their own and other healthcare professions, to ascertain how these may affect readiness for interprofessional learning and professional identity strength	1	0
○ Professional identity and IPI; and identify factors influencing IPI strength	1	0
- Compare the IPI and attitudes towards, perceived facilitators and barriers for, and occurrence of interprofessional treatment for malnutrition and sarcopenia of dietitians and physiotherapists	1	0
- Describe a new conceptual framework or model	1	1
- Evaluate a measurement instrument	2	0
- Examine early professional socialisation experiences of students	1	0
- Explore challenges surrounding IPE and collaborative professional practice related to identities and cultures	1	0
- Explore IPI formation or experiences of/in…		
○ Graduates when transitioning to interprofessional practice	2	0
○ Students during an interprofessional placement	1	0
○ Students during interaction with existing interprofessional teams	1	0
○ Students and clinicians in interprofessional student-clinician interaction, focussing on how they narratively construct identities	1	0

Characteristic	Journal article (*n* = 23)	Book chapter (*n* = 2)
- Identify and describe learning experiences that may support IPI formation	1	0
- Increase conceptual understanding of…		
○ IPI (formation)	3	0
○ IPI in combination with or related to professional identity	1	1
○ Interprofessional socialisation	1	0
- Investigate whether comparative feedback on interprofessional interaction can decrease the degree of profession-based dominance and general dominance in mixed profession groups	1	0
- Investigate whether IPI is a source of intrinsic motivation for IPC	1	0
- Not specified	1	0
Journal		NA
- *Advances in Health Sciences Education*	1	
- *European Journal of Work and Organizational Psychology*	1	
- *International Journal of Environmental Research and Public Health*	1	
- *Journal of Interprofessional Care*	11	
- *Journal of Multidisciplinary Healthcare*	1	
- *Medical Education*	4	
- *Nurse Education Today*	1	
- *PLoS One*	1	
- *Studies in Continuing Education*	1	
- Published as preprint at *Research Square*	1	
Book title	NA	
- *Interprofessional Education and Collaborative Practice: International Approaches at the Micro, Meso, and Macro Levels*		1
- *Teaching Medical Professionalism: Supporting the Development of a Professional Identity*		1
Focus:		
- Empirical study populations encompassing students and/or professionals from varying professions and educational programmes in healthcare	16	0
- Organisational sectors (non-empirical works), e.g., health and social care and (medical/health professions/interprofessional) education	5	2
- Not specified	2	0
Publication type:		
- Conceptual analysis	1	0
- Conceptual analysis combined with critical interpretative synthesis	1	0
- Development of a new conceptual framework or model	1	1
- Literature review not specified	2	1
- Scoping review	3	0
- Commentary	1	0
- Cross-sectional or longitudinal qualitative interview study	5	0
- Cross-sectional or longitudinal quantitative intervention study with or without control group	5	0
- Cross-sectional or longitudinal mixed methods intervention study	2	0
- Evaluation of a (new) measurement instrument	2	0
